# Revealing the mechanisms of warfarin-induced vascular calcification through metabolomics and network toxicology

**DOI:** 10.3389/fphar.2025.1554987

**Published:** 2025-06-09

**Authors:** Zhijiao Zhang, Pengyan Jia, Chunqi Feng, Juan Xu, Jingmin Zhang, Niuniu Bai, Weihong Chen, Weiqi Gao

**Affiliations:** ^1^ Third Hospital of Shanxi Medical University, Shanxi Bethune Hospital, Shanxi Academy of Medical Sciences, Tongji Shanxi Hospital, Taiyuan, China; ^2^ School of Pharmacy, Shanxi Medical University, Taiyuan, China; ^3^ Shanxi Academy of Advanced Research and Innovation (SAARl), Taiyuan, China

**Keywords:** warfarin, vascular calcification, metabolomics, network toxicology, molecular mechanisms

## Abstract

Warfarin is widely used in clinical anticoagulation therapy, but the exact mechanism by which it induces vascular calcification (VC) remains unclear. This study aimed to explore the mechanisms of warfarin-induced VC using metabolomics and network toxicology approaches. Initially, normal rats were orally administered warfarin for 2 weeks, and VC was then assessed by serum biochemistry and histopathology. Subsequently, non-targeted metabolomics was performed to analyze serum metabolite changes. Finally, network toxicology analysis was conducted to identify key targets and signaling pathways associated with warfarin-induced VC, which were further validated using molecular docking, qRT-PCR, and western blot analyses. The results indicated that warfarin induced aortic calcification in rats, and metabolomics identified 32 differential metabolites, mainly involved in pathways such as primary bile acid biosynthesis, steroid hormone biosynthesis, and amino acid metabolism. Network toxicology analysis, molecular docking, and experimental validation showed that warfarin may induce VC by modulating the targets AKT1, TP53, and HSP90AA1, thereby influencing the PI3K-AKT signaling pathway. This study reveals the potential molecular mechanisms underlying warfarin-induced VC, laying a foundation for further mechanistic investigations and providing important insights for the rational clinical application of warfarin.

## Graphical Abstract

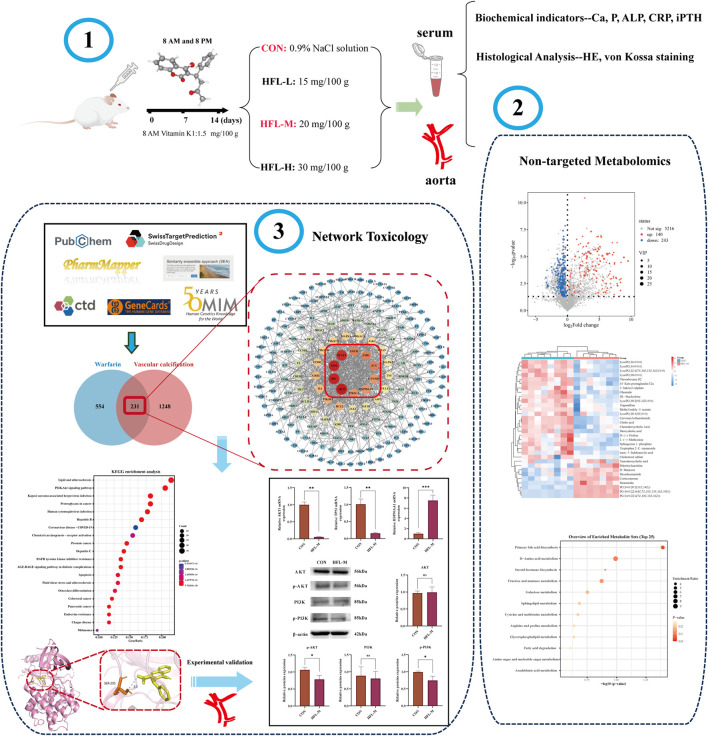

## 1 Introduction

Warfarin, a vitamin K antagonist, effectively reduces the risk of thrombosis by inhibiting the synthesis of vitamin K-dependent clotting factors. It has been widely used in clinical prevention and treatment of thrombotic diseases for many years (Elango et al., 2021). However, recent studies have indicated that long-term use of warfarin may lead to various adverse effects, among which VC has emerged as a significant concern, and related studies have gradually increased (Tantisattamo et al., 2015; Han and O'Neill, 2016; Andrews et al., 2018). Poterucha et al. reported that long-term low-dose or short-term high-dose use of warfarin could induce VC in the coronary arteries and peripheral vascular system, adversely affecting the normal structure and function of blood vessels and potentially exacerbating the development of cardiovascular diseases (Poterucha and Goldhaber, 2016). Additionally, numerous animal studies have demonstrated that oral administration of warfarin can induce VC (McCabe et al., 2013; Van den Bergh et al., 2021; Nasruddin et al., 2024). VC is a common pathological manifestation of atherosclerosis, hypertension, diabetic vascular complications, vascular injury, and chronic kidney disease. It is also an independent risk factor for cardiovascular events and is strongly correlated with poor clinical outcomes (Desai et al., 2018; Lu et al., 2023).

Currently, the pathogenesis of warfarin-induced VC remains unclear. It was once considered a passive, degenerative, and end-stage process of vascular disease, however, recent studies have confirmed that VC results from a highly autonomous regulatory process similar to bone tissue formation (Leopold, 2015; Lanzer et al., 2021). The mechanism underlying this phenomenon is complex and involves multiple factors. Warfarin interferes with the carboxylation of vitamin K-dependent proteins, including matrix Gla protein (MGP), osteocalcin, and bone matrix protein, thereby affecting the activity of proteins involved in bone formation and VC (Siltari and Vapaatalo, 2018; Xiao et al., 2021). Notably, MGP inhibits osteoblast differentiation induced by bone morphogenetic proteins (BMPs) (Zhang and Tang, 2014). Therefore, warfarin may disrupt the balance of factors involved in VC, both directly and indirectly. Furthermore, warfarin-induced VC may be linked to disorders of calcium-phosphorus metabolism and apoptosis (Zhang and Tang, 2014; Demer and Boström, 2015; Siltari and Vapaatalo, 2018; Xiao et al., 2021). Numerous *in vivo* and *in vitro* studies have shown that warfarin may regulate VC through signaling pathways such as Wnt/β-catenin, TG2/β-catenin, EPA/MMP-9, and PXR-BMP2-ALP (Kanai et al., 2011; Beazley et al., 2012; Beazley et al., 2013; Cai et al., 2016; Yu et al., 2019). Despite progress in research on these mechanisms, the precise molecular targets and signaling pathways underlying warfarin-induced VC remain to be fully elucidated.

In recent years, integrating metabolomics and network toxicology techniques has emerged as a novel strategy for investigating the mechanisms of drug toxicity. Metabolomics involves studying changes in disease-related metabolites to identify biomarkers by analyzing the total levels of small molecule metabolites. Understanding the mechanisms of drug action at the metabolic level has become essential in pharmacological research (Newgard, 2017). Network toxicology explores potential drug toxicity mechanisms by constructing “toxic component-target-pathway” networks and investigating key targets and signaling pathways of toxic drugs within the body (Yin et al., 2024).

This study employed serum non-targeted metabolomics to analyze the effects of warfarin on endogenous metabolites and metabolic pathways in normal rats. In conjunction with network toxicology techniques, it identified key target proteins and critical signaling pathways, and elucidated the potential molecular mechanisms of warfarin-induced VC, thereby providing data support for further exploration of these mechanisms.

## 2 Materials and methods

### 2.1 Reagents

Warfarin (purity 98%, batch number RH523030) was purchased from Shanghai Yien Chemical Technology Co., Ltd. (Shanghai, China). Vitamin K1 injection (batch number 2201100211) was purchased from Shanghai Xiandai Hasen Pharmaceutical Co., Ltd. (Shangqiu, China). The Von Kossa Staining Kit (HZ-05282) was acquired from Shanghai Huzhen Industrial Co., Ltd. (Shanghai, China). The rat intact parathyroid hormone (iPTH) ELISA kit (RX301930R) was purchased from Quanzhou Ruixin Biotechnology Co., Ltd. (Quanzhou, China). Calcium Assay Kit (C004-2-1) was purchased from NanJing JianCheng Bioengineering Institute (Nanjing, China). MS-grade acetonitrile, LC-grade formic acid, LC-grade methanol, and the Pierce BCA Protein Assay Kit (23227) were procured from Thermo Fisher Co., Ltd. (MA, United States). PI3K (T40115F), p-PI3K (PC6417), AKT (T55561), and p-AKT (T40067) primary antibodies were purchased from Abmart Shanghai Co., Ltd. (Shanghai, China). β-actin (66009-1-IG) primary antibody was purchased from Proteintech Group, Inc. (Wuhan, China). Enhanced Chemiluminescence (ECL) (BMU102) was purchased from Abbkine Scientific Co., Ltd. (Wuhan, China).

### 2.2 Animal experimentation

Male Sprague-Dawley rats weighing 180 ± 20 g were obtained from Sipei Fu (Beijing) Biotechnology Co., Ltd. (Animal License No.: SCXK-(Jing) 2019-0010). The rats were housed in individual cages at 21°C–25°C with a 12-h light/dark cycle and had unrestricted access to a standard diet and water. The rat VC model was established as previously described (Price et al., 2000). After a 7-day adjustment period, the rats were randomly divided into four groups (n = 8 per group): the control group (CON), the low-dose warfarin group (HFL-L), the medium-dose warfarin group (HFL-M), and the high-dose warfarin group (HFL-H). Twenty-four and 48 hours before the first administration of warfarin, all rats received doses of 1.5 mg of vitamin K1 per 100 g of body weight. Previous studies have indicated that this loading dose of vitamin K is necessary to prevent bleeding during the first week of warfarin treatment (Price et al., 2000). The three administration groups received warfarin by gavage at 8 a.m. and 8 p.m. at doses of 15, 20, and 30 mg/100 g, respectively, along with a concomitant subcutaneous injection of vitamin K1 (1.5 mg/100 g) at 8 a.m. for a duration of 2 weeks. The CON group received the same volume of 0.9% NaCl solution as the administration groups. Throughout the administration period, the body weight of the rats was recorded daily, and their behavioral changes were monitored. After the final administration, all rats were fasted for 12 h with access to water. The following day, the rats were anesthetized, and blood was extracted via retroorbital puncture. Blood samples were allowed to stand at room temperature for 1 h before being centrifuged at 3,000 rpm for 15 min at 4°C. Serum was then prepared and stored at −80°C for subsequent biochemical and metabolomic analyses. Subsequently, the aortas of the rats in each group were rapidly excised and stored at −80°C for further analysis.

### 2.3 Biochemical indicators and vascular calcium content measurements

The levels of calcium (Ca), phosphorus (P), alkaline phosphatase (ALP), and C-reactive protein (CRP) in rat serum were measured using a fully automated biochemical analyzer (Mindray, Shenzhen, China). Serum iPTH levels were quantified by ELISA. Additionally, aortic arch tissue was collected, and vascular calcium content was measured using a calcium assay kit.

### 2.4 Histological analysis

Aortic tissues were fixed in 10% formalin and subsequently embedded in paraffin. They were then cut into 5 µm thick sections for hematoxylin-eosin (HE) and von Kossa staining. The pathological changes in the aorta were observed using a microscope (Nikon, Tokyo, Japan).

### 2.5 Serum non-targeted metabolomics analysis

#### 2.5.1 Preparation of samples

Serum samples from the CON group (n = 8) and HFL-M group (n = 7) were used for non-targeted metabolomic analysis. The serum samples were thawed on ice, and 100 μL of serum was mixed with 400 μL of pre-cooled acetonitrile, vortexed for 30 s, and then centrifuged at 13,000 rpm for 20 min at 4°C. The supernatant was then transferred to a 2 mL tube, concentrated, dried, redissolved in 70 μL of pre-cooled 80% acetonitrile, and centrifuged once under the same conditions as described above. The supernatant was used to prepare the serum sample for testing. Additionally, 5 μL of each sample was collected separately and mixed to create a quality control (QC) sample. LC-MS analyses were performed using an ultra-high-performance liquid chromatography-mass spectrometer (Thermo Fisher Scientific, Waltham, MA, United States).

#### 2.5.2 Conditions for mass spectrometry and chromatography

The liquid chromatography column, Waters Acquity UPLC HSS T3 (1.8 μm, 2.1 mm × 100 mm), was purchased from Waters Corporation, United States. The injection volume was 5 μL, and the flow rate was 0.30 mL⋅min^-1^. The mobile phase consisted of 0.1% formic acid water (A) and 0.1% formic acid-acetonitrile solution (B) with gradient elution. The elution conditions were as follows: 0–2 min, 98% A and 2% B; 3–13 min, 50% A and 50% B; 14–17 min, 40% A and 60% B; 18 min, 10% A and 90% B; 19–20 min, 100% B; 20.5–23 min, 98% A and 2% B.

The ionization method used was heated electrospray ionization (HESI) with the following parameters: a spray voltage of 3,500 V (positive)/3,000 kV (negative); a capillary temperature of 320°C and a heater temperature of 300°C; the sheath gas flow rate was set at 35 arb and the auxiliary gas flow rate at 10 arb. The scanning mode was Full Scan/dd-MS2, with an m/z collection range of 100–1,500 in positive and negative ion switching collection mode. The resolution was MS Full Scan 35,000 FWHM and MS/MS 17,500 FWHM. Additionally, the secondary fragmentation energies for MS were specified as 12.5 eV, 25 eV and 37.5 eV.

#### 2.5.3 Data processing and statistical analysis

The original mass spectrometry data were processed using Compound Discoverer 3.3 software for peak extraction, peak comparison, compound identification, and other data processing operations, resulting in a metabolite peak table. Subsequently, the metabolite peak table data were imported into SIMCA 14.1 (Umetrics, Sweden) software for further analysis. Unsupervised principal component analysis (PCA) was conducted to observe the original dispersion of the samples. Supervised orthogonal partial least squares discrimination analysis (OPLS-DA) was employed to analyze the overall differences between the two groups, obtain the variable importance in projection (VIP), and verify the model’s validity through 200 permutation tests. Differential metabolites were screened based on VIP >1 and *p* < 0.05, while referencing the HMDB (https://hmdb.ca/) and PubChem (https://pubchem.ncbi.nlm.nih.gov/) online databases for verification. Finally, the identified differential metabolites were entered into MetaboAnalyst 6.0 (https://www.metaboanalyst.ca/) for metabolic pathway enrichment analysis.

### 2.6 Network toxicology analysis

#### 2.6.1 Potential targets identification of warfarin and VC

Warfarin targets were predicted using PharmMapper (https://www.lilab-ecust.cn/pharmmapper/), SwissTargetPrediction (http://swisstargetprediction.ch/), the Similarity Ensemble Approach (SEA) (https://sea.bkslab.org/), and the Comparative Toxicogenomics Database (CTD) (https://ctdbase.org/). The targets obtained from the four databases were integrated, and duplicate values were removed to identify the warfarin targets.

The term “vascular calcification” was entered into the GeneCards (http://www.genecards.org/) and OMIM (https://www.omim.org/) databases to search for VC-related targets. VC targets were obtained by removing duplicates from all genes screened previously.

The identified warfarin targets were mapped and compared with the VC targets, and a Venn diagram was created. The common targets represent the potential targets of warfarin-induced VC.

#### 2.6.2 Construction of protein-protein interaction (PPI) networks

The intersecting targets were imported into the STRING database (https://cn.string-db.org/), with the species limited to *Homo sapiens*, a minimum required interaction score set to 0.900, and isolated nodes hidden to construct the PPI network. Subsequently, the visualized PPI network was constructed using Cytoscape 3.10.1 software, followed by topological analysis with the Network Analyzer tool. Finally, the top 10 core targets were selected based on their degree values.

#### 2.6.3 Enrichment analysis

The intersecting targets were imported into the Hiplot platform (https://hiplot.com.cn/home/index.html) for Gene ontology (GO) and Kyoto encyclopedia of genes and genomes (KEGG) enrichment analysis, with the species set to *Homo sapiens* and a significance threshold of *p* < 0.05.

#### 2.6.4 Molecular docking

The three-dimensional (3D) structure of warfarin was obtained from the PubChem database (https://pubchem.ncbi.nlm.nih.gov/), and the crystal structures of the core targets were retrieved from the Protein Data Bank (PDB; https://www.rcsb.org/) database. AutoDock 1.5.7 software (University of Southern California, USC, United States) was utilized to perform the docking of the core proteins with the drug, and the results of the docking were visualized with PyMOL software (Schrödinger, NY, United States).

#### 2.6.5 Quantitative reverse transcription-polymerase chain reaction (qRT-PCR)

Total RNA was extracted from aortic tissues using the SevenFast Total RNA Extraction Kit, and the concentration and purity of the RNA were measured. Subsequently, reverse transcription was performed, followed by qRT-PCR using the SevenFast Two Step RT and qPCR Kit. β-actin was used as the internal reference gene, and the data were analyzed using the 2^−ΔΔCT^ method. The primer sequences are listed in Table 1.

**TABLE 1:** The qRT-PCR primer sequences.

Gene	Primer	Sequence (5′–3′)	Lengths (bp)
β-actin	Forward	CAG​ATG​TGG​ATC​AGC​AAG​CAG​GA	23
	Reverse	CGC​AAC​TAA​GTC​ATA​GTC​CGC​CTA	24
AKT1	Forward	AGA​AGC​AGG​AGG​AGG​AGG​AG	20
	Reverse	CCC​AGC​AGC​TTC​AGG​TAC​TC	20
TP53	Forward	CCA​GCC​AAA​GAA​GAA​ACC​AC	20
	Reverse	CCT​CAT​TCA​GCT​CTC​GGA​AC	20
HSP90AA1	Forward	AGG​AGG​TTG​AGA​CGT​TCG​C	19
	Reverse	AGA​GTT​CGA​TCT​TGT​TTG​TTC​GG	23

#### 2.6.6 Western blotting analysis

The aorta was removed from the −80°C freezer and placed on ice for homogenization to extract total protein. The protein concentration was subsequently measured using a BCA protein assay kit. The protein samples were transferred to PVDF membranes. The membranes were then incubated overnight at 4°C with a dilution of primary antibodies (β-actin 1:2,000, AKT 1:1,000, p-AKT 1:1,000, PI3K 1:1,000, p-PI3K 1:1,000), followed by incubation with a dilution of HRP-conjugated secondary antibodies (1:8,000). Finally, images were captured using a chemiluminescence imaging system (Bio-Rad, United States). The protein bands were analyzed for grayscale values using ImageJ software.

### 2.7 Statistical analysis

Data analysis was performed using GraphPad Prism 9.5.1 software (CA, United States). Comparisons between two groups were made using an unpaired Student’s t-test, while one-way analysis of variance (ANOVA) was conducted for comparisons among multiple groups. Results are expressed as mean ± standard deviation (SD), with statistical significance determined at *p* < 0.05.

## 3 Results

### 3.1 Rat body weight, biochemical, and pathological results

**FIGURE 1:**
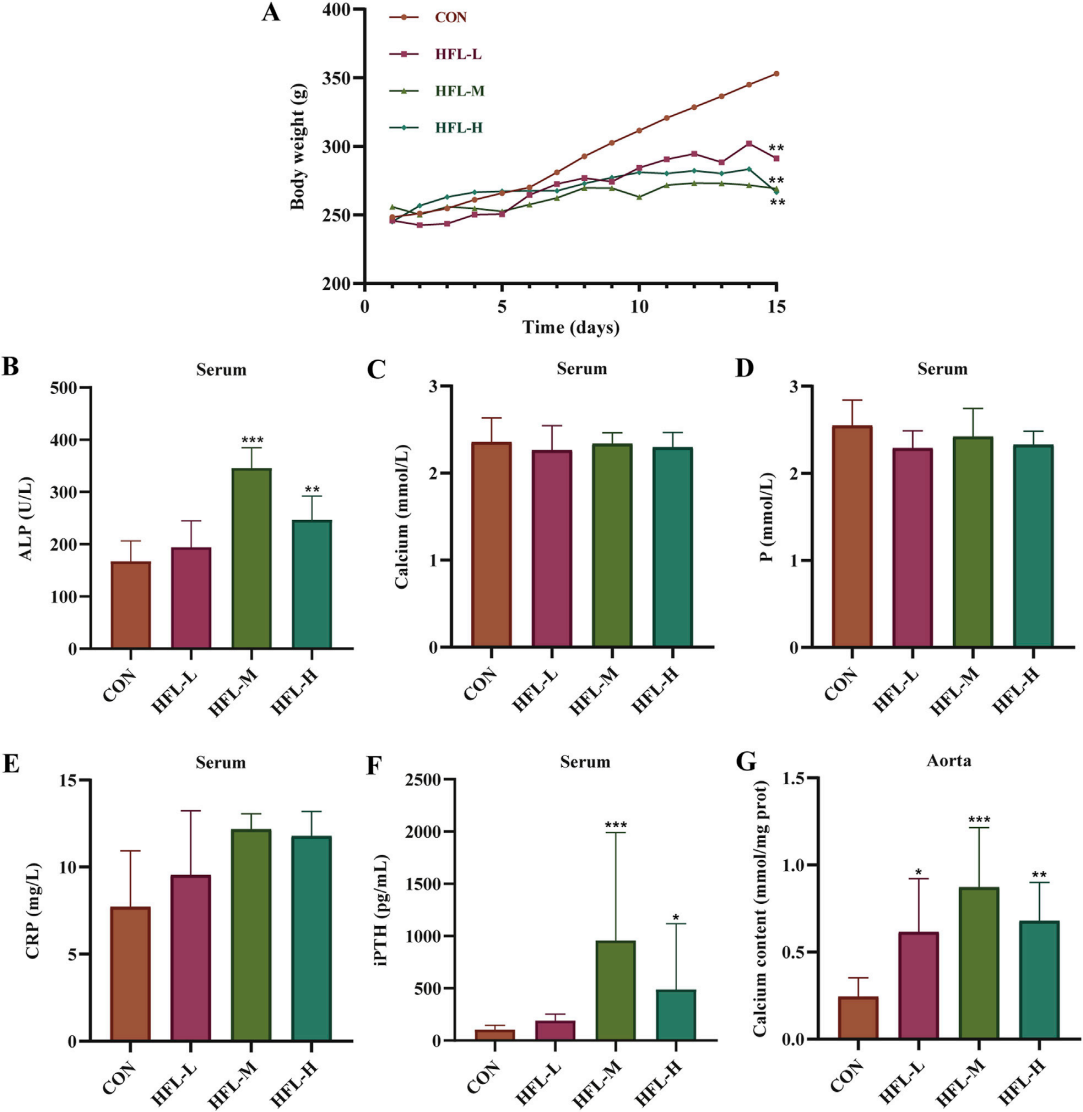
Results of rat body weight and serum biochemical indicators. **(A)** Body weight change. Changes in the levels of serum alkaline phosphatase (ALP) **(B)**, calcium (Ca) **(C)**, phosphorus (P) **(D)**, C-reactive protein (CRP) **(E)**, and intact parathyroid hormone (iPTH) **(F)** in different groups. **(G)** Changes in calcium content within aortic samples of different groups. (Compared with CON group, *p < 0.05, **p < 0.01, ***p < 0.001).

During the 2-week administration period, one rat died in each of the HFL-M and HFL-H groups. Compared to the CON group, the warfarin-administered rats displayed listlessness, reduced activity, and unkempt fur. The body weight of the CON group increased steadily, while the body weight of the warfarin-administered groups also increased, albeit at a slower rate than the CON group (Figure 1A). Serum biochemical analysis revealed significant increases in serum ALP and iPTH levels in the HFL-M and HFL-H groups compared to the CON group (*p* < 0.05), with the HFL-L group showing an upward trend (Figures 1B,F). However, there were no significant differences in serum Ca, P, and CRP levels among all groups, although a trend toward elevated CRP was observed in the administered groups (Figures 1C–E). As shown in Figure 1G, vascular calcium content in the warfarin-administered groups was significantly higher than that in the CON group (*p* < 0.05). These findings suggested the occurrence of VC in the administered rats.

**FIGURE 2:**
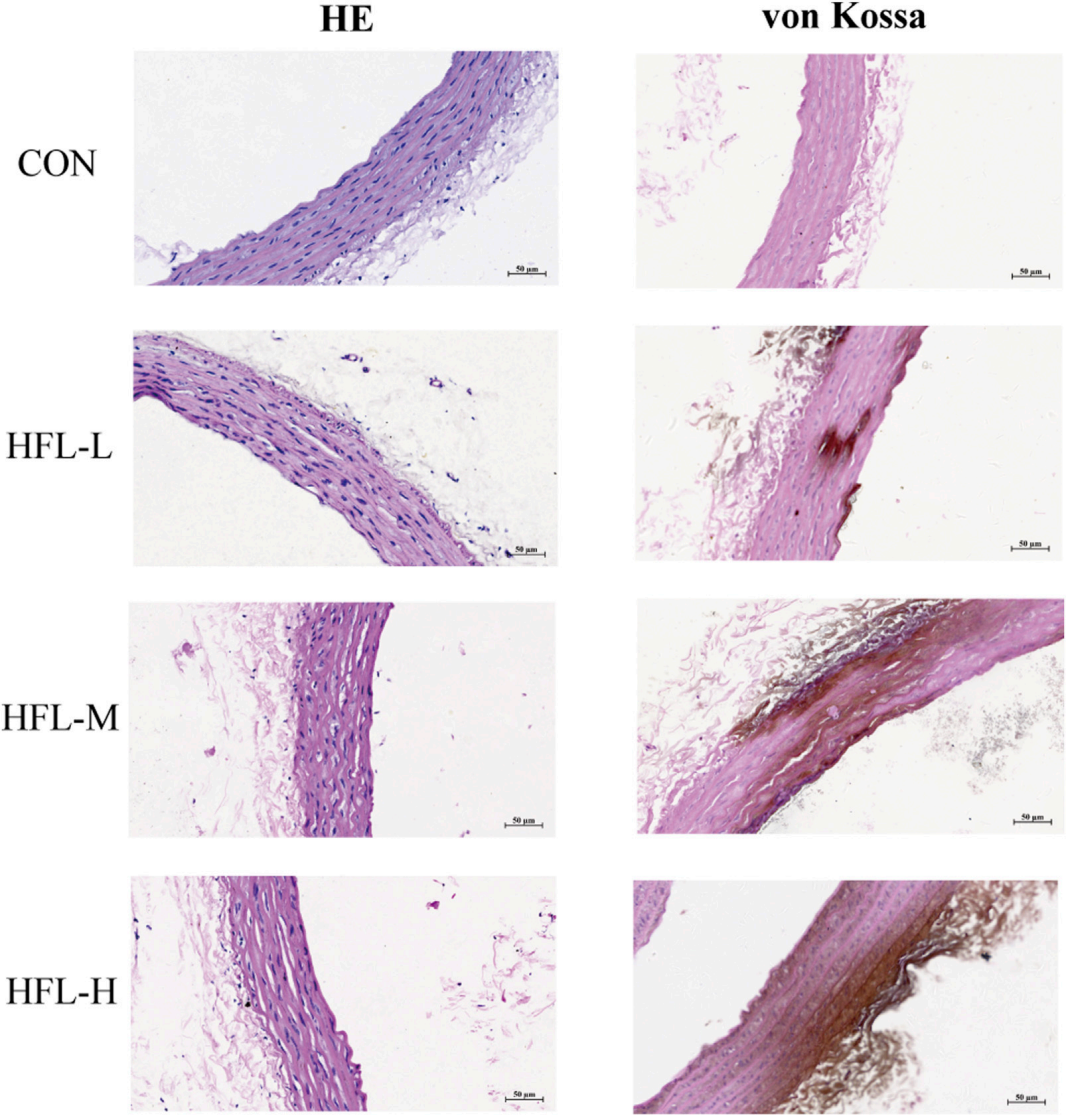
Effects of different doses of warfarin on aortic calcification in rats. Four groups of rats received the following administration regimens. The first row represents the control group (equal volume of 0.9% NaCl solution). The second row represents to the low-dose warfarin group (15 mg/100 g warfarin and 1.5 mg/100 g vitamin K1). The third row represents the medium-dose warfarin group (20 mg/100 g warfarin and 1.5 mg/100 g vitamin K1). The fourth row represents the high-dose warfarin group (30 mg/100 g warfarin and 1.5 mg/100 g vitamin K1). After 2 weeks of administration, longitudinal sections of each aorta were stained with HE (column 1) and von Kossa staining (column 2). HE staining reveals morphological alterations in the aortic tissue, while the brownish-black areas observed in von Kossa staining indicate calcium salt deposition. (scale bar = 50 μm).

Under the influence of warfarin and vitamin K1, HE staining of aortic tissue revealed increased vascular thickness, disorganized vascular smooth muscle cells (VSMCs) arrangement, and disruption of normal vascular structure in the experimental groups. Calcium-specific von Kossa staining showed brownish discoloration in the vasculature of the administered groups, indicating calcium salt deposition, a hallmark of VC (Figure 2). These histopathological findings further confirmed that warfarin induced VC.

In summary, significant differences were observed in several indices between the HFL-M group and the CON group, indicating that VC was more pronounced in the HFL-M group. Therefore, rats in the HFL-M group were selected as subjects for the follow-up study to investigate the mechanism of warfarin-induced VC.

### 3.2 Non-targeted metabolomics

#### 3.2.1 Multivariate data analysis

**FIGURE 3:**
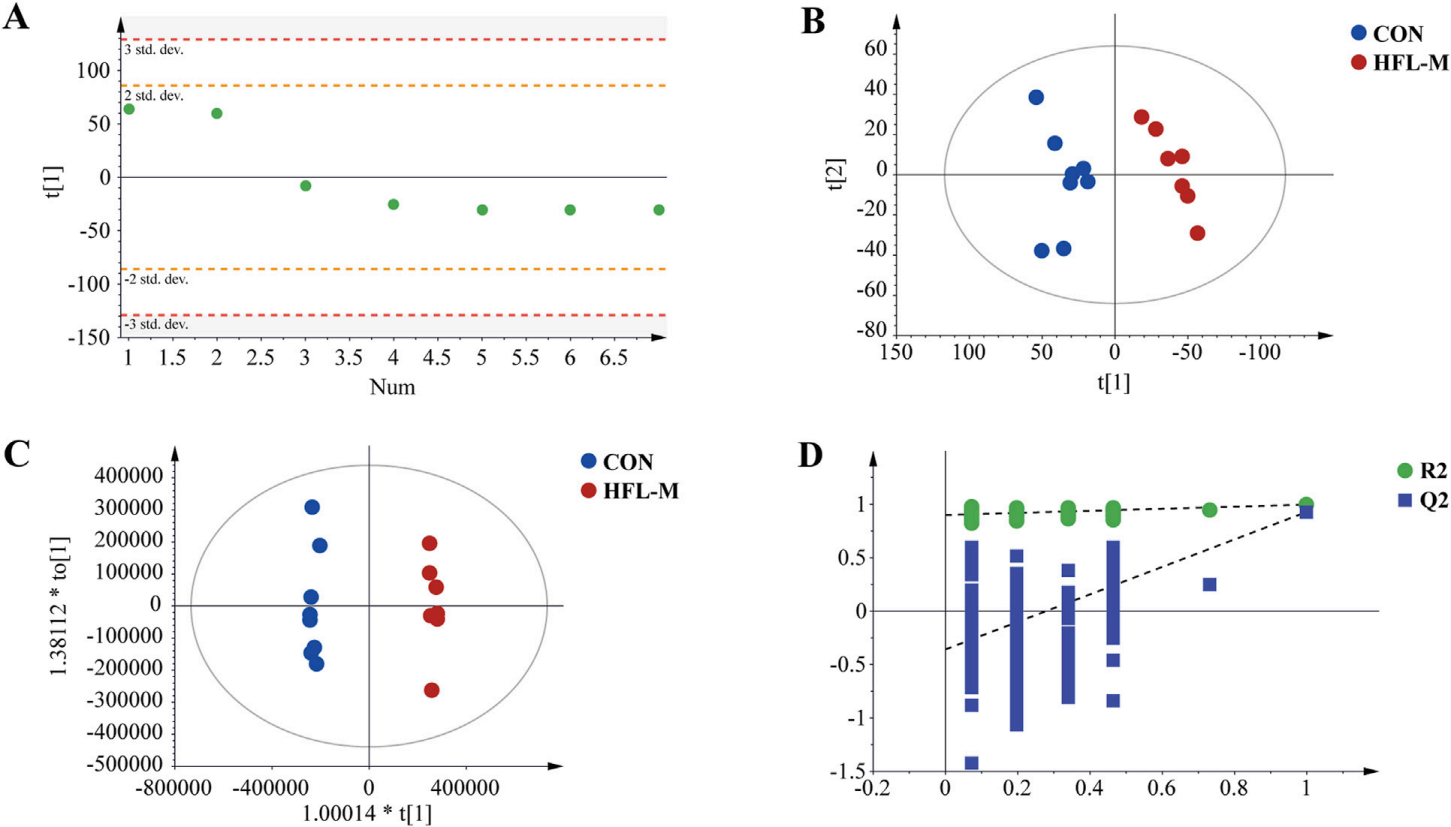
Metabolomic multivariate statistical analysis. **(A)** PCA score plot of the QC samples. **(B)** PCA score plot of the CON and HFL-M groups. **(C)** OPLS-DA score plot of the CON and HFL-M groups. **(D)** The 200-permutation tests of OPLS-DA score plot between the CON and HFL-M groups.

First, PCA was applied to analyze the QC samples and assess their dispersion. The results shown in Figure 3A, indicated that the QC samples fell within the 2 SD range and the 95% confidence interval. This suggested that the QC samples were consistent and that the analytical instrument was stable. Additionally, the PCA score plot indicated that the metabolites were significantly separated between the CON and HFL-M groups (Figure 3B). These findings suggest that warfarin administration induces significant changes in the serum metabolic profiles of the rats. To further analyze the differences between groups, OPLS-DA analysis was employed, and 200 permutation tests was conducted to assess the reliability of the model. The results (Figures 3C,D) revealed an even more pronounced separation trend between the two groups, and the intercept of the regression line of the Q2 of the serum sample on the Y-axis was <0, indicating that the model did not overfit and was effective and reliable.

#### 3.2.2 Identification of differential metabolites

**FIGURE 4:**
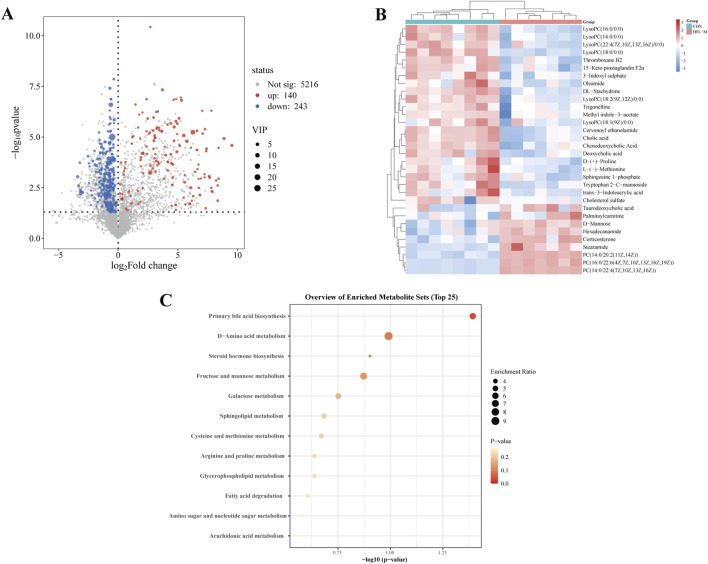
Analysis of differential metabolites in non-targeted metabolomics. **(A)** Volcano plot of the CON and HFL-M groups. **(B)** Heat map of serum metabolites in the CON and HFL-M groups. **(C)** KEGG analysis of metabolic pathways.

Screening for differential metabolites associated with warfarin-induced VC, using the criteria of VIP >1 and *p* < 0.05, identified a total of 383 differential metabolites between the CON and HFL-M groups (Figure 4A). By combining the mass spectrometry information of the compounds, 32 differential metabolites were ultimately identified, as shown in Table 2. A heatmap was created to display the intensity levels of the differential metabolites between the two groups (Figure 4B).

**TABLE 2:** Summary of differential metabolites in the serum of HFL-M and CON rats.

No.	Metabolites	Formula	RT (min)	m/z	Ion Mode	Change Trend (HFL-M/CON)
1	D-Mannose	C_6_H_12_O_6_	0.874	203.0526	M + Na	↑**
2	D-(+)-Proline	C_5_H_9_NO_2_	0.973	116.0708	M + H	↓*
3	Trigonelline	C_7_H_7_NO_2_	0.974	138.0550	M + H	↓*
4	DL-Stachydrine	C_7_H_13_NO_2_	1.018	144.1020	M + H	↓***
5	L-(−)-Methionine	C_5_H_11_NO_2_S	1.134	150.0583	M + H	↓*
6	Tryptophan 2-C-mannoside	C_17_H_22_N_2_O_7_	4.584	367.1500	M + H	↓*
7	trans-3-Indoleacrylic acid	C_11_H_9_NO_2_	4.630	188.0707	M + H	↓*
8	3-Indoxyl sulphate	C_8_H_7_NO_4_S	4.786	212.0023	M − H	↓**
9	Methyl indole-3-acetate	C_11_H_11_NO_2_	5.681	190.0863	M + H	↓**
10	15-Keto prostaglandin F2α	C_20_H_32_O_5_	5.732	335.2215	M + H	↓**
11	Thromboxane B2	C_20_H_34_O_6_	5.740	369.2281	M − H	↓***
12	Taurodeoxycholic acid	C_26_H_45_NO_6_S	6.021	498.2895	M − H	↑*
13	Corticosterone	C_21_H_30_O_4_	6.223	347.2216	M + H	↑***
14	Cervonoyl ethanolamide	C_24_H_36_O_3_	7.294	373.2737	M + H	↓**
15	Cholic acid	C_24_H_40_O_5_	7.295	407.2802	M − H	↓**
16	Chenodeoxycholic acid	C_24_H_40_O_4_	7.681	437.2908	M − H	↓***
17	Sphingosine 1-phosphate	C_18_H_38_NO_5_P	9.824	380.2559	M + H	↓**
18	LysoPC(14:0/0:0)	C_22_H_46_NO_7_P	10.188	468.3087	M + H	↓**
19	Deoxycholic acid	C_24_H_40_O_4_	12.171	391.2854	M − H	↓**
20	LysoPC(18:2 (9Z,12Z)/0:0)	C_26_H_50_NO_7_P	13.435	520.3403	M + H	↓***
21	PC(14:0/22:4 (7Z,10Z,13Z,16Z))	C_44_H_80_NO_8_P	16.273	782.5691	M + H	↑***
22	PC(14:0/20:2 (11Z,14Z))	C_42_H_80_NO_8_P	16.275	758.5692	M + H	↑***
23	PC(16:0/22:6 (4Z,7Z,10Z,13Z,16Z,19Z))	C_46_H_80_NO_8_P	16.285	806.5688	M + H	↑***
24	LysoPC(16:0/0:0)	C_24_H_50_NO_7_P	16.479	496.3401	M + H	↓***
25	LysoPC(18:1 (9Z)/0:0)	C_26_H_52_NO_7_P	17.746	522.3560	M + H	↓*
26	Palmitoylcarnitine	C_23_H_45_NO_4_	18.045	400.3421	M + H	↑*
27	LysoPC(22:4 (7Z,10Z,13Z,16Z)/0:0)	C_30_H_54_NO_7_P	18.735	572.3711	M + H	↓**
28	Cholesterol sulfate	C_27_H_46_O_4_S	19.491	465.3045	M − H	↓*
29	Stearamide	C_18_H_37_NO	19.616	284.2947	M + H	↑**
30	Hexadecanamide	C_16_H_33_NO	19.879	256.2633	M + H	↑**
31	LysoPC(18:0/0:0)	C_26_H_54_NO_7_P	20.004	524.3715	M + H	↓***
32	Oleamide	C_18_H_35_NO	21.057	282.2791	M + H	↓*

↑***, significantly increased compared with Con group (*p* < 0.001); ↑**, significantly increased compared with Con group (*p* < 0.01); ↑*, significantly increased compared with Con group (*p* < 0.05). ↓***, significantly decreased compared with Con group (*p* < 0.001); ↓**, significantly decreased compared with Con group (*p* < 0.01); ↓*, significantly decreased compared with Con group (*p* < 0.05).

#### 3.2.3 Analysis of metabolic pathway

The 32 differential metabolites were imported into MetaboAnalyst 6.0 for KEGG metabolic pathway analysis. As shown in Figure 4C, the occurrence of warfarin-induced VC may be closely related to pathways such as primary bile acid biosynthesis, D-amino acid metabolism, steroid hormone biosynthesis, sphingolipid metabolism, glycerophospholipid metabolism, and cysteine and methionine metabolism and so on.

### 3.3 Network toxicology analysis

#### 3.3.1 Potential targets prediction of warfarin and VC

Network toxicology approaches were employed to investigate the mechanisms of warfarin-induced VC at the protein level. Warfarin targets were obtained from four databases: 102 from SwissTargetPrediction, 275 from PharmMapper, 20 from SEA, and 460 from CTD. After removing duplicates, a total of 785 unique warfarin targets were identified. Using the search term “vascular calcification”, the relevant targets identified included 104 from OMIM and 1,401 from GeneCards. After merging the targets from both databases and removing duplicates, a total of 1,479 unique VC-related genes were identified.

#### 3.3.2 Construction of PPI network

**FIGURE 5:**
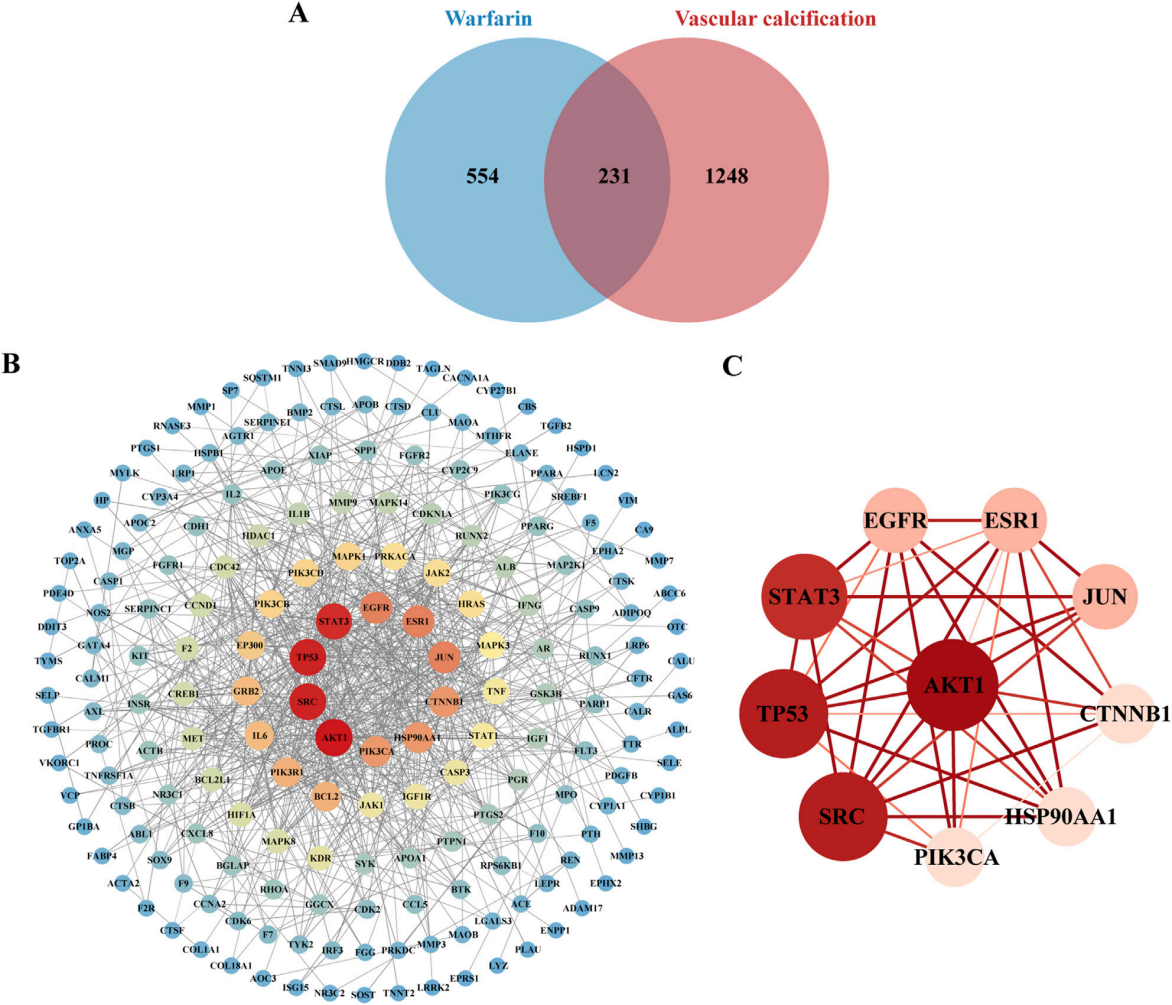
Key targets of warfarin-induced VC. **(A)** Venn diagram showing the overlapping targets of warfarin and VC. **(B)** Visualization of the PPI network using Cytoscape. **(C)** PPI network construction of core targets.

Analysis of a Venn diagram identified 231 common targets of warfarin and VC (Figure 5A). A PPI network was created for the common targets and visualized using Cytoscape 3.9.1 (Figure 5B). Based on the degree values, the top 10 core targets of warfarin-induced VC were selected: AKT1, SRC, TP53, STAT3, EGFR, ESR1, JUN, CTNNB1, HSP90AA1, and PIK3CA (Figure 5C).

#### 3.3.3 Enrichment result analysis

**FIGURE 6:**
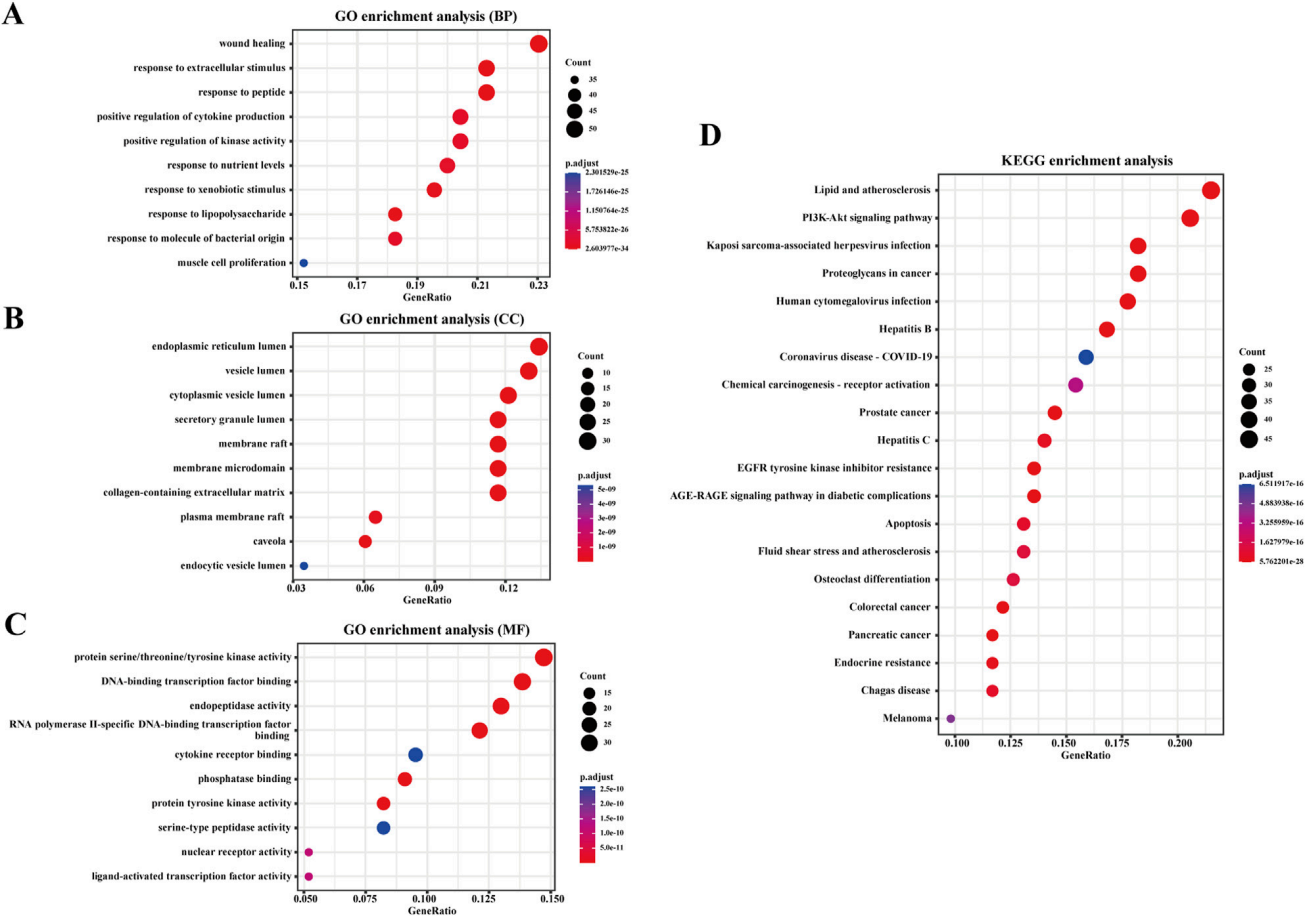
Enrichment analysis. **(A)** GO enrichment analysis based on BP, **(B)** CC, **(C)** and MF. **(D)** KEGG enrichment analysis for the overlapping targets of warfarin and VC. The analysis is based on predicted gene targets from public databases.

GO and KEGG enrichment analyses were performed on the common targets to identify the potential pathways involved in warfarin-induced VC. The GO enrichment analysis encompassed three categories: biological processes (BP), cellular components (CC), and molecular functions (MF). The top ten entries for BP, CC, and MF are presented in Figures 6A–C. The BP primarily included wound healing, response to extracellular stimuli, and response to peptides, among others. The CC primarily included the endoplasmic reticulum lumen, vesicle lumen, and cytoplasmic vesicle lumen, among others. The MF primarily included protein serine/threonine/tyrosine kinase activity, DNA-binding transcription factor binding, and endopeptidase activity, among others. The KEGG enrichment analysis revealed that warfarin-induced VC was closely associated with the PI3K-Akt signaling pathway (Figure 6D).

#### 3.3.4 Molecular docking

**FIGURE 7:**
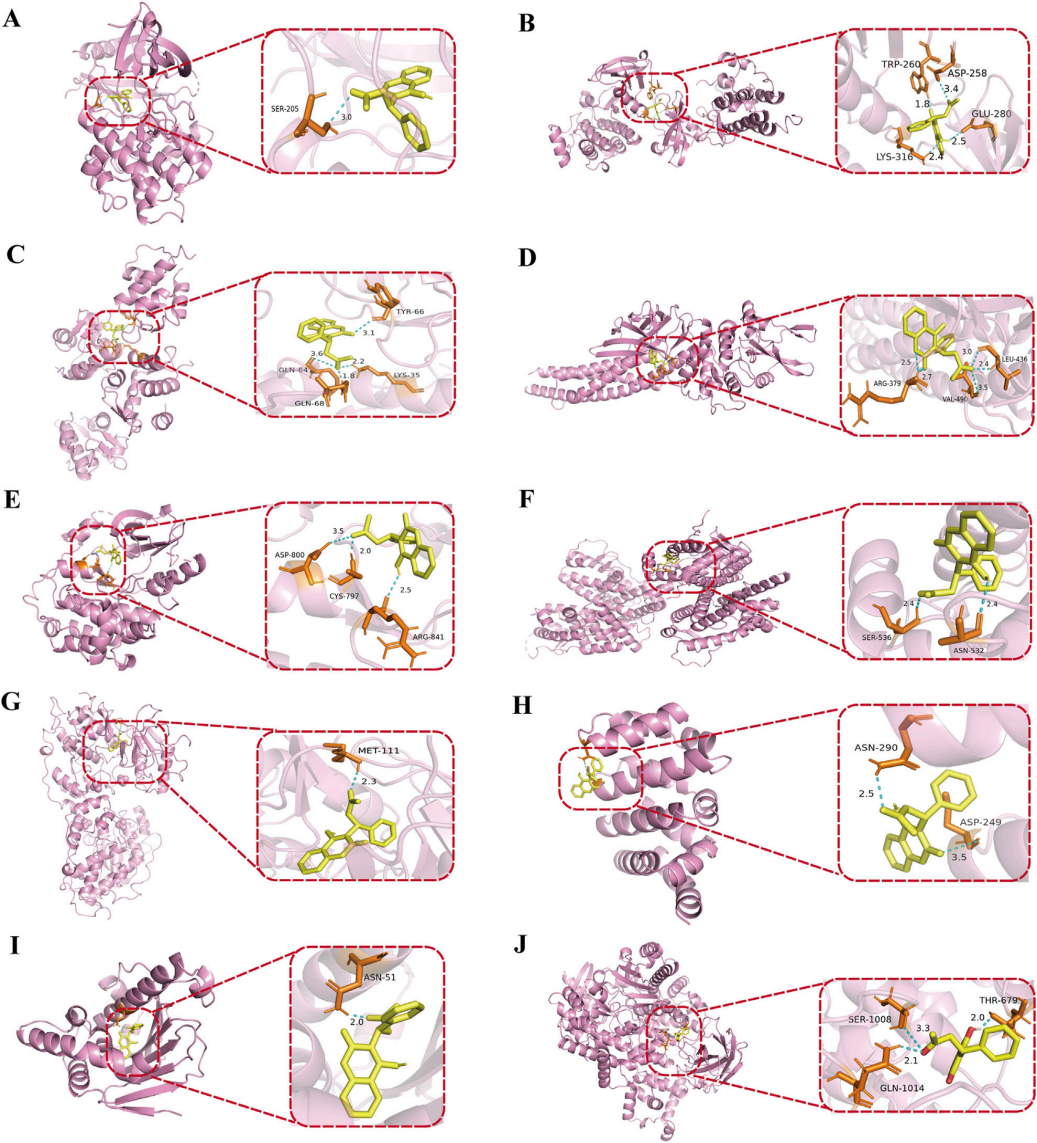
Molecular docking of warfarin with core proteins. **(A)** warfarin with AKT1, **(B)** warfarin with SRC, **(C)** warfarin with TP53, **(D)** warfarin with STAT3, **(E)** warfarin with EGFR, **(F)** warfarin with ESR1, **(G)** warfarin with JUN, **(H)** warfarin with CTNNB1, **(I)** warfarin with HSP90AA1, **(J)** warfarin with PIK3CA. The protein structures were obtained from the PDB: AKT1 (PDB ID: 3QKL), SRC (PDB ID: 1FMK), TP53 (PDB ID: 4IBU), STAT3 (PDB ID: 6NJS), EGFR (PDB ID: 5CNO), ESR1 (PDB ID: 7RRY), JUN (PDB ID: 4D3Q), CTNNB1 (PDB ID: 7AFW), HSP90AA1 (PDB ID: 1UY6), and PIK3CA (PDB ID: 8EXL). In the visualizations, yellow represents warfarin, pink represents the target proteins bound to warfarin, and dashed lines indicate hydrogen bonds.

To further validate the binding sites and affinity between warfarin and the core targets of VC, molecular docking was conducted using AutoDock. The receptors selected for docking were AKT1, SRC, TP53, STAT3, EGFR, ESR1, JUN, CTNNB1, HSP90AA1, and PIK3CA, with warfarin acting as the docking ligand. The docking results were visualized using PyMOL software (Figures 7A–J). The interaction strength between warfarin and the target proteins was assessed based on binding energy, with lower values indicating greater complex stability and stronger binding affinity. The binding energies presented in Table 3 demonstrated stronger interactions between warfarin and AKT1, TP53, and HSP90AA1.

**TABLE 3:** The binding energy of molecular docking.

Drug	Target	Binding energy (kcal/mol)
Warfarin	AKT1	−9.0
	SRC	−7.2
	TP53	−8.2
	STAT3	−6.5
	EGFR	−7.5
	ESR1	−6.7
	JUN	−7.7
	CTNNB1	−6.8
	HSP90AA1	−9.4
	PIK3CA	−8.0

#### 3.3.5 qRT-PCR and western blotting analysis verification

**FIGURE 8:**
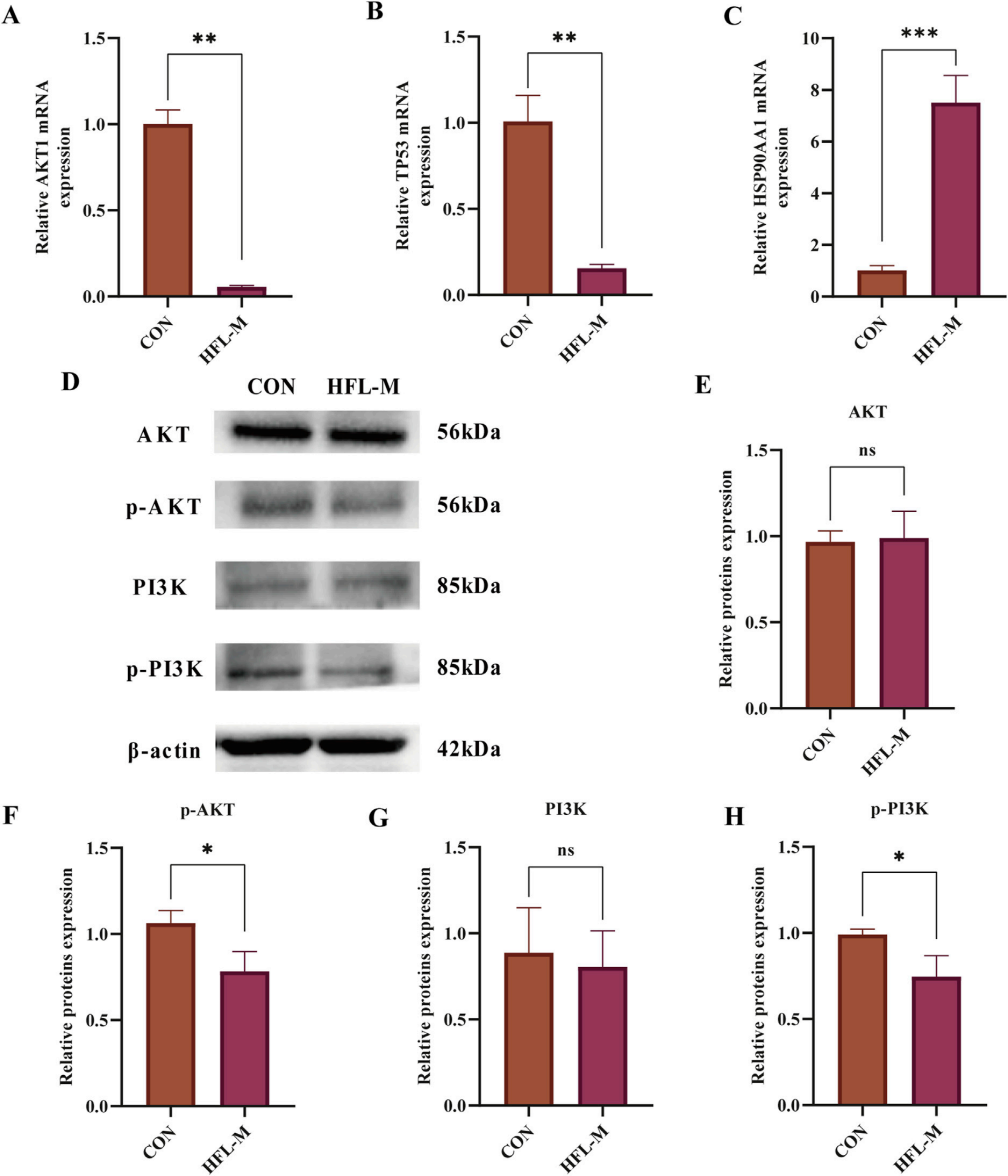
The effects of warfarin on the expression of core protein in the aorta of rats. **(A–C)** qRT-PCR analysis AKT1, TP53 and HSP90AA1 mRNA levels in aorta tissues of rats (n = 3). **(D–H)** Western blot analysis of AKT and PI3K protein expression in aorta tissues of rats (n = 3). Results are expressed as the mean ± SD. *p <, **p < 0.01, ***p < 0.001 vs. the control group.

To validate the findings from network toxicology and molecular docking, qRT-PCR and Western blotting analysis were performed to assess the expression of core proteins and key pathways. The qRT-PCR results indicated that, compared to the CON group, the mRNA levels of AKT1 and TP53 in the aorta of rats in the HFL-M group were significantly decreased (*p* < 0.01), whereas the mRNA level of HSP90AA1 was significantly increased (*p* < 0.001), as illustrated in Figures 8A–C. Western blotting analysis revealed that the protein expressions of PI3K and AKT in the HFL-M group were not significantly different from those in the CON group, whereas the expressions of p-PI3K and p-AKT were significantly decreased (*p* < 0.05) (Figures 8D–H).

## 4 Discussion

The complex mechanisms underlying VC lead to the lack of significantly effective treatments, and once calcifications occur, reversal becomes very difficult. Therefore, there is an urgent need for high-dimensional analysis to identify novel therapeutic targets to address warfarin-induced VC. In this study, we firstly constructed a VC model in rats through different doses of warfarin combined with vitamin K1 intervention, and systematically evaluated the validity of the model from the perspectives of serum biochemical indexes, vascular calcium content, and histopathology. Serum biochemical results showed that there were no significant abnormalities in serum levels of Ca, P, and CRP among the rats groups, but the CRP levels in the warfarin-administered group showed an increasing trend. Ca and P are important minerals in the human body. Traditionally, VC has been believed to be closely related to hypercalcemia and hyperphosphatemia, yet some studies have pointed out that VC is not correlated with serum levels of Ca and P (Schurgers et al., 2007; Sun et al., 2020). CRP is an acute-phase reactive protein, and although it can reflect certain calcification-associated inflammatory responses, it serves only as a weaker predictor of VC (Avula et al., 2025). In addition, serum levels of ALP and iPTH were significantly elevated in the HFL-M and HFL-H groups. Serum ALP is currently recognized as an important marker of bone formation and VC, while iPTH, a peptide hormone secreted by the parathyroid gland, when abnormally elevated, not only accelerates the release of calcium phosphate salts but also promotes the formation of calcium phosphate crystals, with its level being positively correlated with the degree of calcification (Bosworth et al., 2013; Nizet et al., 2020). Then combined with the results of vascular calcium content detection, we found that the vascular calcium content of the warfarin-administered group was significantly higher than that of the CON group, and the aortic pathological sections revealed disordered arrangement of VSMCs and pronounced calcium salt deposition in the warfarin-administered rats. These findings suggest that VC occurred in rats in the low, medium, and high dose warfarin groups, though the relationship did not follow a typical dose-response pattern. Among them, the degree of VC in the HFL-L group was relatively mild, and the degree of calcification in the HFL-M group was more significant than that in the HFL-H group, which was different from the expected linear dose-effect relationship. We speculate that once warfarin dosage reaches a certain threshold, its pro-calcific effect may approach saturation, resulting in a plateau phase between the HFL-M and HFL-H dose ranges, where the extent of calcification no longer increases with higher doses. This phenomenon suggests that warfarin-induced VC may have a complex dose-window effect, and its specific mechanism and critical dose value need to be further verified through subsequent studies such as expanding the sample size and adding gradient dose groups.

Based on these observations, we selected rat samples from the HFL-M and CON groups for metabolomics and network toxicology analysis to investigate the mechanisms of warfarin-induced VC from a holistic perspective, emphasizing a “multi-target, multi-pathway” strategy. Through metabolomic analysis, we found that a total of 32 differential metabolites in the serum of warfarin-induced VC rats were significantly changed, including 9 upregulated and 23 downregulated metabolites. Heatmap analysis of the differential metabolites indicated that metabolite levels were relatively close within the groups, while distinct color differences between the CON and HFL-M groups indicated significant metabolic changes between them. Further pathway enrichment analysis revealed that these differential metabolites primarily focused on primary bile acid biosynthesis, steroid hormone biosynthesis, and amino acid metabolism.

Bile acids, as the end products of cholesterol metabolism in the liver, are the primary pathway of eliminating excess cholesterol and maintaining systemic cholesterol homeostasis. Impaired cholesterol excretion can promote lipid deposition and lead to VC. In the warfarin-administered group, four bile acids showed significant alterations, among which cholic acid, chenodeoxycholic acid and deoxycholic acid were significantly downregulated, and taurodeoxycholic acid was significantly upregulated. Cholic acid, deoxycholic acid, and chenodeoxycholic acid are activators of the farnesoid X receptor (FXR), with their effects increasing in that order. In contrast, taurodeoxycholic acid acts as an antagonist of FXR (Li et al., 2007). It has been shown that FXR is a bile acid nuclear receptor, and its activation can regulate pro-inflammatory mediators, reduce triglyceride and cholesterol levels, diminish macrophage inflammation and lipid uptake, and also inhibit the proliferation and migration of VSMCs to stabilize plaques in an FXR-SHP-dependent manner (Miyazaki-Anzai et al., 2010). This suggests that warfarin may induce VC by reducing the levels of cholic acid, chenodeoxycholic acid, and deoxycholic acid, while increasing the level of taurodeoxycholic acid, thereby inhibiting FXR activation and promoting the occurrence of VC. Previous studies have found that ABCC6 deficiency causes calcification to cause significant reductions in circulating cholic acid, chenodeoxycholic acid, and deoxycholic acid concentrations in mice, while upregulation of taurodeoxycholic acid is also associated with the formation of calcified plaques in mice, which is consistent with the results of this study (Brampton et al., 2021; Wang et al., 2024). Thus, these results suggest that warfarin may induce VC by modulating the bile acid network.

Existing studies have demonstrated that steroid hormone biosynthesis also plays an important role in the pathogenesis of VC. In this study, we observed significant changes in two steroid hormones, including cholesterol sulfate (CS) and corticosterone. CS is an important steroid sulfate in the human body and has been recognized as a new biomarker of atherosclerosis. We observed a significant downregulation of CS in the serum of warfarin-administered rats. This finding coincides with the hypothesis proposed by Seneff et al., which suggests that CS deficiency may be a key pathological factor underlying atherosclerosis (Seneff et al., 2015). Corticosterone is a physiological glucocorticoid. Our results showed an upregulation of corticosterone levels in the serum of warfarin-administered rats. Studies have reported that there are mineralocorticoid receptors (MR) in calcified vascular cells, and corticosterone can reduce cell viability and stimulate VSMCs apoptosis by activating MR. The exposure of apoptotic bodies to phosphatidylserine on the outer membrane can produce potential calcium-binding sites suitable for hydroxyapatite deposition, thereby inducing VC (Jaffe et al., 2007). *In vitro* studies on mice VSMCs calcification by Zhu et al. also confirmed that corticosterone promotes calcification by directly activating MR in VSMCs (Zhu et al., 2016).

Furthermore, the amino acid metabolic pathways involved in this study mainly included D-amino acid metabolism, cysteine and methionine metabolism. Among them, proline and methionine in the serum of warfarin-administered group were significantly downregulated. Proline, as a key amino acid for collagen synthesis, its metabolic abnormalities may affect the stability and elasticity of vascular wall (Li and Wu, 2018). To the best of our knowledge, there have been no animal studies on the effect of proline on arterial calcification, but proline has been found to inhibit calcium and phosphate-induced apoptosis of VSMCs in cell culture (Shimomura et al., 2014). Chen et al. also found that patients with a coronary artery calcium (CAC) score >100 had lower serum proline levels (Chen et al., 2025). Methionine is an essential amino acid for the human body, which can be oxidized into methionine sulfoxide to directly target reactive oxygen species (ROS), effectively remove the accumulation of ROS in the body, alleviate oxidative stress, and inhibit osteogenic differentiation (Li et al., 2019). A study using a 3D *in vitro* calcification model demonstrated that methionine significantly reduced the percentage of calcium per unit area, suggesting a potential anti-calcification effect (Coutts et al., 2023).

Subsequently, we conducted network toxicology analysis to further explore the potential targets of warfarin-induced VC, and screened out a total of 231 relevant targets. Using the Network Analyze tool, AKT1, SRC, TP53, STAT3, EGFR, ESR1, JUN, CTNNB1, HSP90AA1, and PIK3CA were identified as core genes, and the signaling pathway involved was mainly PI3K-AKT signaling pathway. Then molecular docking revealed strong binding affinities between warfarin and AKT1, TP53, and HSP90AA1, and the three key targets and key pathway were further verified by *in vivo* experiments. The results showed that warfarin downregulated the mRNA levels of AKT1, TP53 and the protein levels of p-PI3K and p-AKT in rat aorta, while upregulating the mRNA levels of HSP90AA1.

AKT1 (also known as Protein Kinase B, PKB) is a serine/threonine kinase that belongs to the AKT family and is involved in various cellular signaling processes such as cell growth, proliferation, migration, and angiogenesis. It has been proposed as a promising target for the treatment of cardiovascular diseases (Kumar et al., 2025). Previous studies have found that AKT1 alleviates apoptosis and abnormal proliferation by inhibiting forkhead box O3 (FoxO3a) and its downstream gene apoptotic protease activating factor 1 (Apaf1) in VSMCs (Tucka et al., 2014). Xiao et al. reported that ubiquitin-specific protease 47 gene knockout significantly reduced the expression levels of AKT1 and MGP in rat aortic VSMCs, thereby inducing osteoblastic differentiation (Xiao et al., 2022). In a high inorganic calcium/phosphate-induced mice calcification model, a reduction in AKT1 activation in calcified aortic tissue was also observed (Li et al., 2021). This is consistent with the role of AKT1 in inhibiting VC. TP53 (tumor protein 53) is an important tumor suppressor gene that encodes a transcription factor involved in regulating the cell cycle, cell apoptosis, and cellular stress responses. It protects cells from apoptosis by directly inducing antioxidant genes such as glutathione peroxidase 1 and aldehyde dehydrogenase 4, or by indirectly regulating proteins associated with glucose and glutamine metabolism to reduce intracellular ROS levels (Wu et al., 2017). Liu et al. found that DNA topoisomerase II inhibitors mainly induce TP53 expression to activate miR-203-3p, thereby inactivating the BMP2 pathway and alleviating VC (Liu et al., 2018). Similarly, Ni et al. observed a significant decrease in TP53 mRNA expression in phosphate-induced calcified VSMCs (Ni et al., 2024). HSP90AA1 (heat shock protein 90α) is a molecular chaperone protein that is mainly expressed under stress conditions and is involved in protein folding, stabilizing and degrading, and signal transduction. Ding et al. found that the serum HSP90AA1 levels in patients with atherosclerotic vascular disease were significantly increased, which promoted the expression of Runx2, a marker of endothelial cell calcification, by activating low-density lipoprotein receptor-related protein 1 (Ding et al., 2024). In addition, HSP90AA1 can also activate the Wnt/β-catenin signaling pathway to promote osteogenic phenotypic transformation (Lee et al., 2022). It can be seen that the upregulation of HSP90AA1 is likely to be related to the occurrence of VC.

Phosphoinositide 3-kinase-protein kinase B (PI3K-AKT) signaling pathway is a key signaling transduction pathway widely present in various cells and plays a central role in regulating cell proliferation, differentiation, and growth. In recent years, numerous studies have revealed that the PI3K-AKT signaling pathway serves as a crucial bridge in regulating VC (Pan et al., 2023). Inhibition of the expression of PI3K-AKT signaling in VSMCs significantly reduces the expression of the calcification inhibitor MGP (PonnUnited Statesmy et al., 2018). Meanwhile, inhibition of the PI3K-AKT signaling axis can induce ferroptosis, and activation of VSMCs ferroptosis further promotes VC (Lu et al., 2024; Yang et al., 2025). Bai et al. pointed out that the PI3K/AKT signaling pathway is inhibited in VSMCs induced by calcification medium, while FTI-277, SET8 can prevent apoptosis and inhibit mineral deposition by upregulating PI3K/AKT signaling (Bai et al., 2022). Our results also confirm that warfarin may promote the occurrence of VC by inhibiting the PI3K-AKT pathway. These findings highlight the importance of the PI3K-AKT signaling pathway in influencing VC.

This study has certain limitations, particularly the lack of in-depth targeted metabolomics analysis of key metabolic markers and pathways identified through serum non-targeted metabolomics. Consequently, future research will focus on the pathways identified in this study to further explore the mechanisms underlying warfarin-induced VC.

## 5 Conclusion

In summary, this study investigated the mechanisms of warfarin-induced VC through metabolomics, network toxicology, and experimental validation. Our results suggest that warfarin may target AKT1, TP53, and HSP90AA1, inhibit the PI3K-AKT signaling pathway, and further affect pathways such as primary bile acid biosynthesis, steroid hormone biosynthesis, and amino acid metabolism, ultimately leading to VC. Therefore, this study offers insights and theoretical justification for a comprehensive analysis of the mechanisms underlying warfarin-induced VC.

## Data Availability

The data presented in the study are deposited in the MetaboLights repository, accession number MTBLS12511.

## Abbreviations

VC, Vascular Calcification; MGP, Matrix Gla Protein; BMP, Bone Morphogenetic Protein; Ca, Calcium; P, Phosphorus; ALP, Alkaline Phosphatase; CRP, C-reactive Protein; iPTH, intact Parathyroid Hormone; HE, Hematoxylin-Eosin; QC, Quality Control; PCA, Principal Component Analysis; OPLS-DA, Orthogonal Partial Least Squares Discrimination Analysis; VIP, Variable Importance in Projection; SEA, Similarity Ensemble Approach; CTD, Comparative Toxicogenomics Database; PPI, Protein-Protein Interaction; GO, Gene Ontology; KEGG, Kyoto Encyclopedia of Genes and Genomes; qRT-PCR, Quantitative Reverse Transcription-Polymerase Chain Reaction; SD, Standard Deviation; VSMC, Vascular Smooth Muscle Cell; BP, Biological Processe; CC, Cellular Component; MF, Molecular Function; CAC, Coronary Artery Calcification; FXR, Farnesoid X Receptor; CS, Cholesterol Sulfate; MR, Mineralocorticoid Receptor; TP53, Tumor Protein 53; HSP90AA1, Heat Shock Protein 90 α.
